# Altered Value Coding in the Ventromedial Prefrontal Cortex in Healthy Older Adults

**DOI:** 10.3389/fnagi.2016.00210

**Published:** 2016-08-31

**Authors:** Jing Yu, Loreen Mamerow, Xu Lei, Lei Fang, Rui Mata

**Affiliations:** ^1^Faculty of Psychology, Southwest UniversityChongqing, China; ^2^Department for Cognitive and Decision Sciences, University of BaselBasel, Switzerland; ^3^Key Laboratory of Mental Health, Institute of Psychology, Chinese Academy of SciencesBeijing, China; ^4^Faculty of Medicine, Southeast UniversityNanjing, China; ^5^Max Planck Institute for Human DevelopmentBerlin, Germany

**Keywords:** aging, decision making, ventromedial prefrontal cortex, anterior insula, Balloon Analogue Risk Task

## Abstract

Previous work suggests that aging is associated with changes in risk taking but less is known about their underlying neural basis, such as the potential age differences in the neural processing of value and risk. The goal of the present study was to investigate adult age differences in functional neural responses in a naturalistic risk-taking task. Twenty-six young adults and 27 healthy older adults completed the Balloon Analogue Risk Task while undergoing functional magnetic resonance imaging. Young and older adults showed similar overt risk-taking behavior. Group comparison of neural activity in response to risky vs. control stimuli revealed similar patterns of activation in the bilateral striatum, anterior insula (AI) and ventromedial prefrontal cortex (vmPFC). Group comparison of parametrically modulated activity in response to continued pumping similarly revealed comparable results for both age groups in the AI and, potentially, the striatum, yet differences emerged for regional activity in the vmPFC. At whole brain level, insular, striatal and vmPFC activation was predictive of behavioral risk taking for young but not older adults. The current results are interpreted and discussed as preserved neural tracking of risk and reward in the AI and striatum, respectively, but altered value coding in the vmPFC in the two age groups. The latter finding points toward older adults exhibiting differential vmPFC-related integration and value coding. Furthermore, neural activation holds differential predictive validity for behavioral risk taking in young and older adults.

## Introduction

Aging is associated with changes in cognition, emotion, and motivation that have important consequences for decision making (Tymula et al., 2013; Samanez-Larkin and Knutson, 2015; Schiebener and Brand, 2015). For example, recent meta-analyses suggest that aging is associated with changes in a variety of risky choice tasks (Mata et al., 2011; Best and Charness, 2015). But what are the potential mechanisms underlying age-related changes in dealing with risk and uncertainty? In our work, we aim to contribute to the understanding of possible mechanisms underlying age differences in risk taking by investigating young and older adults’ neural activations associated with a well-known risk-taking task, the Balloon Analogue Risk Task (BART; Lejuez et al., 2002). The BART is a popular and useful tool for measuring cognitive and affective mechanisms underlying risk-taking behavior (Lejuez et al., 2002; Schonberg et al., 2012), thus also representing a promising tool to uncover sources of age differences in cognitive and motivational components on decision making.

Participants in the BART are asked to pump up a balloon as much as they like, which, although leading to increased accumulation of (monetary) gains with each pump, simultaneously increases the probability of the balloon exploding (Lejuez et al., 2002). Thus, risk on the BART refers to the probability of an explosion resulting in the loss of all accumulated gains in a trial. The structure of the task captures not only participants’ valuation of possible gains and losses simultaneously but also affective processes that could arise as a consequence of the increasing tension and uncertainty associated with additional pumps on a given balloon. As such, the BART mimics the risk–reward trade-off as well as the sequential process that characterizes decisions in many natural environments (Schonberg et al., 2011; Pleskac and Hertwig, 2014). Importantly, the task may have some predictive validity for real-life impulsive or risk-taking behavior, such as drug use, delinquency, gambling, and risky sexual behaviors (Lejuez et al., 2003, 2004; Aklin et al., 2005; Hunt et al., 2005).

The BART has found wide application in the field of behavioral as well as neural research, yielding a backdrop of findings for the current work. Specifically, previous neuroimaging studies have identified a set of key brain regions as being differentially involved in this task, including the ventromedial prefrontal cortex (vmPFC), dorsal lateral prefrontal cortex (dlPFC), anterior cingulate cortex (ACC), anterior insula (AI), striatum, and the midbrain (Rao et al., 2008, 2014; Chiu et al., 2012; Lighthall et al., 2012; Schonberg et al., 2012; Kohno et al., 2013; Telzer et al., 2013; Helfinstein et al., 2014). All of these areas have been implicated—in some form or another and with more or less specificity—in the construction, representation and storage of subjective value (for reviews, see Glimcher, 2010; Levy and Glimcher, 2012; Bartra et al., 2013; Clithero and Rangel, 2014). Specifically, although striatal and frontal activation patterns are widely recognized as key regions for value-based judgment and decision making, insular activation appears to be more prevalent for paradigms in which decision making extends beyond purely deliberative and into affective processing, including loss anticipation and harm avoidance (Paulus et al., 2003; Knutson and Bossaerts, 2007; Preuschoff et al., 2008; Mohr et al., 2010a; Bartra et al., 2013). Further work relevant to risk taking on the BART pertains to the neural correlates of uncertainty, with previous work implicating the right AI in the tracking of uncertainty (e.g., Volz et al., 2003). However, considering that uncertainty often implies the possibility of loss or harm, it is somewhat unclear whether the covariation between insular activity and uncertainty reflects the tracking of the abstract (mathematical) or affective component of uncertainty.

Of particular interest to this study are previous results obtained with the BART that identified decreasing vmPFC activation as a neural correlate of risk taking (Schonberg et al., 2012; Rao et al., 2014). For several decision-making tasks, vmPFC activity has been implicated in the representation of subjective value; that is, representing a signal that reflects the outcome of an integration of reward, risk (uncertainty), and potentially also affective evaluation (Kim et al., 2010; Rangel and Hare, 2010; Rushworth et al., 2011; Levy and Glimcher, 2012; Bartra et al., 2013). Some have proposed that the vmPFC is a critical substrate for information integration which triggers secondary emotional responses that help guide advantageous decision-making (Bechara and Damasio, 2005; Levin et al., 2012). Considering these previous studies and theoretical models of decision making, vmPFC-related activation in the BART could be representative of an integrative function of the vmPFC, coding the decreasing subjective value of additional pumping over time by integrating the potential gains with the increasing probability of loss (i.e., explosion). Taken together, the properties of the BART that make it a comparatively valid behavioral measure of risk taking—where risk is understood not only as outcome variability but also as exposure to potential loss—are mirrored in neural activity patterns. Previous work that adopted the BART in conjunction with findings from other paradigms provide some insight into the possible functional roles of different neural regions on the BART, including the coding of loss, reward, uncertainty, and integrated (subjective) value, each of which could be affected by cognitive and neural changes due to aging.

With regards to the computational drivers of age-related behavioral and neural differences in risk taking, it has been proposed that aging may be associated with difficulties in learning or representing the subjective (integrated) value of options, which can conceptually be thought of as arising from noisy representations due to low signal-to-noise ratio of information processing (Li and Rieckmann, 2014). For example, older adults typically show difficulties in learning the utility of options from probabilistic feedback, possibly due to age-related declines in neuromodulator systems that help form value representations (Li et al., 2007; Mohr et al., 2010b; Eppinger et al., 2011; Chowdhury et al., 2013). In one study, Samanez-Larkin et al. (2014) showed age-related reduction in the frontal representation of reward prediction error for paradigms involving feedback-based learning, but no such differences for the representation of reward magnitude. Moreover, several studies have shown differences in vmPFC-related reward and value signals as a function of age (Baena et al., 2010; Mohr et al., 2010b; Eppinger et al., 2013; Halfmann et al., 2016), leading to the suggestion that increasing variability in vmPFC signaling accounts for differences in performance (Rogalsky et al., 2012; Halfmann et al., 2016). The notion of increasingly varied neural responses, both with regards to inter-individual and intra-individual variability, is not limited to the vmPFC and related functions, but has already been found in other neural areas implicated in decision-making processes aversively affected by age (Li et al., 2007; Samanez-Larkin et al., 2010). Moreover, affective changes over the human lifespan may impact on decisions under risk, both behaviorally (Huang et al., 2013; Shao and Lee, 2014) and neurally (Shao and Lee, 2014). Taken together, multiple pathways are implicated in accounting for age-related changes in decision making (under risk), and several—such as altered information integration, feedback-based learning, or changes in affective responses to stimuli, choices and their outcomes—could play a role in leading to age differences in the BART. A few behavioral studies have used the BART to investigate adult age differences in risk taking. However, the results of extant comparisons of young and older adults using the BART are inconsistent; although two found that older adults were somewhat less risk-seeking relative to young adults (Henninger et al., 2010; Rolison et al., 2012), another found the opposite (Cavanagh et al., 2012). Gaining a better understanding of the different neural components underlying age differences in the BART could be helpful in predicting when young and older adults differ in risk taking.

The goal of the present study was to investigate adult age differences in neural signals of risky decision making on the BART, a paradigm that captures the perceptible escalating tension between risk and reward not evident in other paradigms (e.g., described lotteries). Thus, we were interested in using the BART to compare young and older adults’ neural signatures of risky decision making and establish whether differences arise in areas previously implicated in processes subsumed in the concept of risk taking, specifically the notion of harm avoidance and tracking of potential losses in the insula and the representation of utility (i.e., value) in the vmPFC. Moreover, we were particularly interested in assessing whether signals originating in the insular cortex or the vmPFC are similarly predictive of individual differences in behavioral outcomes (i.e., risky choices). We thus hoped to contribute to the challenge of uncovering possible age differences in decision making under risk, and eventually the dissociation of drivers of age-related differences such as the processing of reward, risk and subjective value.

## Materials and Methods

### Participants

Twenty-six young and 27 older adults were recruited for the present study. Young adults were students of Southwest University, China, and older adults were recruited from communities in or near Southwest University. One young and three older adults were excluded due to excessive head movement during scanning (see below for exclusion procedure). In addition, one older adult was excluded for cashing out all reward balloons after just one pump. Forty-eight healthy right-handed participants were included in the final analyses, 25 young adults (11 male, mean age: 21.0 ± 1.6 years, age range: 18–24 years) and 23 older adults (eight male, mean age: 65.3 ± 5.3 years, age range: 60–79 years). Participants had no prior history of stroke, neurological or psychiatric disorder, and all older participants were independent community-dwelling adults whose Mini-Mental State Examination (MMSE; Folstein et al., 1975) scores were above 26 (mean score: 29.2 ± 1.2). Participants received 60 CNY (ca. 10 USD) for participation in the study, with the opportunity to earn up to an additional 15 CNY (ca. 2.5 USD) based on performance in the decision task. All participants provided written informed consent and the study was approved by the Institutional Review Board of the Brain Imaging Center, Southwest University, China.

### Materials and Procedures

Participants completed a variant of the BART inside the MRI scanner (for further information on previous uses of the BART, see Lejuez et al., 2002; Schonberg et al., 2012). Prior to entering the scanner, participants were given instructions and completed a short practice trial. They were told that their goal was to maximize their scores in the task to increase their final payment. Participants could inflate a balloon on each of a number of trials by pressing a “pump” button. Each pump could earn participants 0.1 CNY (ca.0.02 USD); however, if the balloon exploded, they would lose the money accumulated in that trial. In order to avoid the explosion, participants could “cashout” the money at any point and secure their money by adding it to the “bank.” There were three balloon types in the task, two reward balloons and one control balloon. Control balloons were gray balloons, which did not explode but also had no monetary value. Participants were simply asked to pump up the gray balloons until they disappeared from the screen. The two reward balloons could lead to monetary gains but differed in the maximum number of pumps that they could receive, thus creating a distinction between high- and low-capacity balloons. We used the two balloons as proxies for low- and high-risk conditions in contrast to no risk for the control balloon in order to examine whether behavioral and/or neural differences would emerge as a function of risk level and also whether this effect would be subject to age differences. Participants were not provided with any information about the differences between high- and low-capacity balloons but could in principle keep track of the two different types because they were assigned a different color, red or blue, with color assigned to each balloon type being counterbalanced between participants. The probability of the balloons exploding (or disappearing from the screen, in the case of the control balloons) was *p*(explosion) = 1/(*maximum*-*pumps*), with a maximum of 12, 20, 16 for the low-capacity, high-capacity, and control balloons, respectively. The order of presentation of the balloons was randomized. The task was self-paced, therefore the number of balloons varied between participants in the fixed-duration 10-min scanning run. The interval between pumps varied randomly between 1 and 2 s, and the interval between trials (balloons) varied between 1 and 12 s, with a mean of 4.5 s.

### Behavioral Analysis

We calculated the average number of pumps for cashout balloons (i.e., adjusted pumps), as is typically done in the BART literature in order to limit analyses to balloons for which the final number of pumps was not capped by an explosion (Lejuez et al., 2002). We also calculated the average number of reward trials, proportion of cashout trials, and average reaction time for each pump. We performed a 2 (age: young vs. older) × 2 (balloon: high- vs. low-capacity) repeated measures ANOVA on adjusted pumps, and conducted one-way ANOVAs on the number of reward balloons, proportion of cashout trials, and mean reaction time to estimate age differences. Statistical analyses of behavioral data were performed using SPSS 20.0 (IBM Corporation, Somers, NY, USA).

### Image Acquisition

Participants were scanned at the Brain Imaging Center in Southwest University using a 3.0 T Siemens Tim Trio MRI system (Erlangen, Germany). For each participant, functional echo planar image data were collected using the following parameters: time repetition (TR) = 2000 ms, time echo (TE) = 30 ms, flip angle = 90°, field of view (FOV) = 200 mm × 200 mm, 33 axial slices, slice thickness = 3.0 mm, gap = 0.6 mm, acquisition matrix = 64 × 64, in-plane resolution = 3.125 × 3.125, and 200 volumes. High-resolution, three-dimensional T1-weighted structural images were acquired for each participant, with the following parameters: 176 slices, acquisition matrix = 256 × 256, voxel size = 1 mm × 1 mm × 1 mm, TR = 1900 ms, TE = 2.2 ms, and flip angle = 9°.

### Image Preprocessing

Data preprocessing was performed using the Statistical Parametric Mapping program^1^ (SPM8). First, the difference in acquisition time between slices was corrected, followed by a rigid-body correction for head motion. Participants included in the present study had less than 3.0 mm maximum translation and 3.0° rotation head motion throughout the scan. For normalization, we used a study-specific template created using unified segmentation and diffeomorphic image registration (DARTEL, Diffeomorphic Anatomical Registration using Exponential Lie Algebra; Ashburner, 2007). First, each subject’s image was segmented into gray matter, white matter, and cerebral spinal fluid probabilistic images. The segmented gray-matter images were then normalized to Montreal Neurological Institute (MNI) space as defined by SPM8. DARTEL represents better localization of functional magnetic resonance imaging (fMRI) activity than does the optimized normalization procedure, by treating the brain template as a deformable probability density map, comparing the signal intensities of each voxel for every brain (Leshikar and Duarte, 2014). The resulting normalized images were then spatially smoothed using a 6 mm full-width half-maximum (FWHM) kernel to decrease spatial noise.

### fMRI Analysis

Analysis of the functional MRI data was carried out in three steps. First, neural activity was modeled using the general linear model in a similar fashion to previous studies (Schonberg et al., 2012) with a high-pass filter of 1/128 Hz. In the general linear model analysis, two regressors for pumps were included: (1) Pumps_Average_, capturing average activity across all pumps, and (2) Pumps_Parametric_, capturing parametrically modulated activity by sequentially increasing the number of pumps within each trial. These two regressors were also implemented for the control balloons (Control_Average_ and Control_Parametric_). Because we found no significant differences between the activities elicited by the low- and high-capacity balloons, the two experimental balloon types were collapsed and a single regressor was used to model both types of trials. In order to remove visual and motor effects unrelated to risk and reward processing, we contrasted the reward pumps to those in the control condition (Pumps_Average_ vs. Control_Average_ and Pumps_Parametric_ vs. Control_Parametric_). To control for the potential confounding effects of head movement, six motion parameters (three translation and three rotation parameters) were entered into the GLM as regressors of no interest. The resulting activation patterns were labeled positive effects for a BOLD signal that was higher for reward than for control balloons when contrasted, whereas higher BOLD for control vs. reward balloons was taken to indicate a negative effect. Two-sample *t*-tests were computed to determine age group differences, specifically to examine BOLD signal differences between groups in each contrast to observe the influence of age on neural activity related to risk taking. Moreover, in order to illustrate the age differences on “Pumps_Average_ vs. Control_Average_” contrast more clearly, we conducted the two-sample *t*-tests masked by a positive effect map and a negative effect map, respectively, to observe the age differences on the positive effect regions and negative effect regions separately. The positive effect mask is a binary mask, which was generated from the combination of young and older age groups’ positive effect map on “Pumps_Average_ vs. Control_Average_” contrast after correction, and the negative effect mask was generated likewise.

Whole-brain regression analyses were performed in order to identify brain regions that correlated with participants’ risk-taking behavior. We examined the correlation between each individual’s neural activity during Pumps_Parametric_ vs. Control_Parametric_ and his/her mean number of adjusted pumps. The individual difference analysis in the form of whole brain regression was conducted both across groups and by age group.

In addition to the whole-brain regression analysis, region of interest (ROI) analyses were adopted; these allowed us to test for the neural–behavioral association in specific brain regions that might not have been captured after correcting for multiple comparisons at whole-brain level. ROIs were created as 4 mm radius spherical regions covering the bilateral AI and striatum, respectively, and an 8 mm radius spherical region in the vmPFC. The center coordinates for the ROI masks (vmPFC [2 46 -8]; left AI [-36 20 -6]; right AI [40 22 -6]; left striatum [-12 4 2]; right striatum [12 10 -2]) were defined based on a recent meta-analysis examining neural correlates of subjective value (Bartra et al., 2013). In addition to using published coordinates to build ROI masks, center coordinates derived from the current sample (peak coordinate from Pumps_Parametric_ vs. Control_Parametric_ across age groups, vmPFC [-15 39 -12]; left AI [-33 24 3]; right AI [39 21 6]; left caudate [-12 6 9]; right caudate [9 3 9]) were used in secondary analyses aimed at testing the reliability of the results. These supplementary analyses also included spheres of different sizes, with sphere radii ranging from 3 to 10 mm, covering 1-mm increments between the lower and upper bound. Pearson’s correlation analysis was performed to evaluate the relation between activation in the bilateral AI and the vmPFC (activation from parametric contrast on increasing number of pumps) and an individual’s behavioral performance (i.e., mean adjusted pumps).

Functional magnetic resonance imaging analyses were examined at a threshold corrected for multiple comparisons (corrected by the false discovery rate, FDR, *p* < 0.05). All coordinates are reported in MNI format. Anatomical labels of neural regions were obtained by importing the resulting statistical parametric maps into xjview^2^.

## Results

### Behavioral Results

**FIGURE 1:**
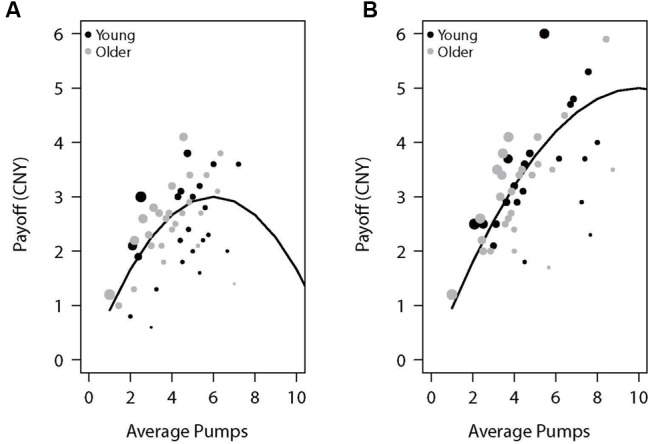
**Payoff as a function of average pumps for the **(A)** low-capacity and **(B)** high-capacity reward balloons.** The lines represent the expected value of the specific average pumps across 10 trials of each balloon type (the average number of trials experienced by participants). Each dot represents a participant, with its diameter being a function of the proportion of cashout trials.

**Table 1** presents the average adjusted pumps, proportion of cashout trials, and other BART variables separately for young and older adults. We also plotted the performance as a function of adjusted number of pumps for each participant in the two reward balloons (**Figure 1**). For individuals’ distribution of pumps for low- and high-risk balloons, see the (Supplementary Figure S1). As expected, participants behaved adaptively by pumping more in the high-capacity relative to the low-capacity balloon but most participants showed risk-averse behavior in the sense of pumping less than the expected value maximizing amount. Concerning age differences, as can be seen in **Table 1** and **Figure 1**, older adults were more likely to cash their earnings relative to young adults, yet this tendency did not translate into a significantly lower number of pumps or earnings for either balloon type. A 2 (age: young vs. older) × 2 (balloon: high-capacity vs. low-capacity) mixed-model ANOVA on adjusted pumps did not find age differences [*F*(1,46) = 0.82, *p* = 0.371] but a significant effect of balloon [*F*(1,46) = 8.17, *p* < 0.01] with more pumps being observed for the high-capacity relative to the low-capacity balloon. The interaction between age and balloon type was also not significant [*F*(1,46) = 0.01, *p* = 0.944]. These results suggest that both young and older participants learned to differentiate between the two balloons despite not having been explicitly informed about the differences. Moreover, average reaction times were larger for older adults (**Table 1**).

**Table 1:** Behavioral results in young and older adults Groups (M ± SD).

Outcome	Young adults	Older adults	*F*	*p*
Mean adjusted pumps	4.82 ± 1.55	4.43 ± 1.56	0.76	0.388
Number of reward balloons experienced	20.92 ± 2.41	19.65 ± 3.24	2.39	0.129
Proportion of cashout trials	0.61 ± 0.12	0.70 ± 0.13	7.38	<0.010
Mean pump RT (ms)	521.10 ± 88.70	815.13 ± 341.18	17.33	<0.001

In sum, although young and older adults did not differ in average adjusted pumps, older adults had more cashout trials than young adults, possibly indicating more risk-averse behavior in older relative to young participants. We now turn to the issue of potential age differences in neural activations in the BART.

### fMRI Results

In what follows, we present three sets of fMRI analyses. First, we report comparisons between average neural activity associated with pumping on experimental (i.e., balloons that were associated with monetary gains/losses) relative to control balloons (i.e., balloons that were not associated with any monetary gains/losses) for young and older adults, as well as any differences between the two groups. This comparison allowed us to capture reward/loss processes and age differences therein while subtracting activation due to attentional or motor processes that were of no interest to the current research. Second, we report parametric analyses of the neural activity of experimental relative to control balloons as a function of the number of pumps administered on a given trial. The rationale for this second set of analyses is similar to the one above but the pump-by-pump analysis provides a window into the processing of risk and reward as it unfolds over the course of a single trial. Finally, we report individual difference analyses that link neural activation of specific regions of interest to behavioral levels of risk taking. These latter analyses clarify the functional role of specific neural activations and whether these are differentially informative regarding individual and age differences in risk-taking behavior.

#### Neural Activity: Average Effects

**FIGURE 2:**
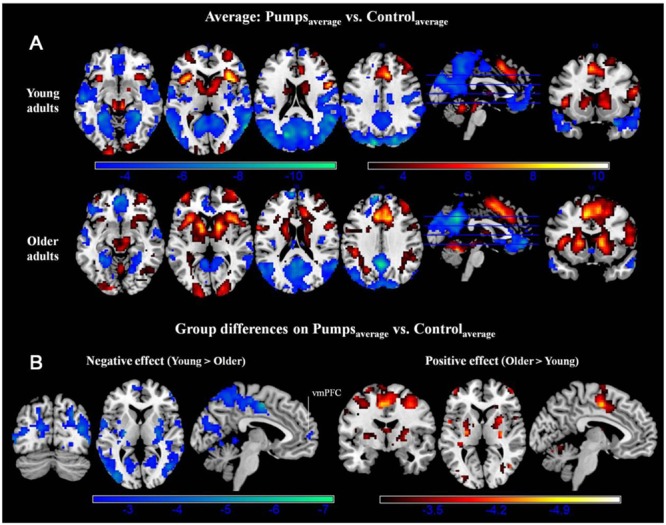
**(A)** Average activity during pumps in the young and older age group. The red scale represents Pumps_Average_ > Control_Average_ and the blue scale represents Control_Average_ > Pumps_Average_. **(B)** Age group differences for average neural activity during pumps. Blue patterns represent neural regions in which negative effects were larger for the young compared with the older age group. Red patterns represent neural regions in which the positive effect was larger for older than for young adults, *p* < 0.05, corrected (scale represents the range of *t*-values). No brain regions were discovered for which (1) young adults had larger positive effects relative to older adults or (2) older adults had larger negative effects than young adults.

A whole-brain contrast revealed widespread neural activity for the reward vs. control pumps contrast. Specifically, both young and older adults displayed positive effects (i.e., Pumps_Average_ > Control_Average_) in the bilateral AI, striatum (caudate and putamen), dorsal ACC, superior frontal cortex and the visual cortex (**Figure 2A**, Red; **Tables 2** and **3**, Average: Pumps_Average_ > Control_Average_). These areas have been identified in previous studies of the BART (Rao et al., 2008; Schonberg et al., 2012) and similar decision tasks (Mohr et al., 2010a; Wu et al., 2012; Bartra et al., 2013) as being related to reward and risk processing. Moreover, both age groups displayed negative effects (i.e., Control_Average_ > Pumps_Average_) in the inferior frontal gyrus, middle temporal gyrus, precuneus, and the vmPFC (**Figure 2A**, Blue; **Tables 2 and 3**, Average: Control_Average_ > Pumps_Average_). In particular, activity in the vmPFC has been shown to correlate with valuation in various decision-making tasks (Levy and Glimcher, 2012; Bartra et al., 2013), including the BART (Schonberg et al., 2012; Rao et al., 2014).

**Table 2:** Significant clusters of activation in young adults.

Region	L/R/B	*X*	*Y*	*Z*	*t*-value	Cluster size
**Average**						
**Pumps_Average_ > Control_Average_**						
Insula	R	39	18	3	10.28	109
Insula	L	-33	21	3	8.66	142
Superior frontal gyrus	R	30	57	15	7.12	143
Middle frontal gyrus	L	-33	54	9	5.05	92
Cingulate	B	9	27	30	9.94	181
Calcarine	R	18	-96	-3	6.82	55
Middle occipital gyrus	L	-18	-96	-3	7.06	67
**Control_Average_ > Pumps_Average_**						
Temporal lobe, Parietal lobe, Precuneus	B	21	-78	39	11.40	15251
vmPFC	B	36	42	-18	7.27	478
**Parametric**						
**Pumps_Parametric_ > Control_Parametric_**						
Insula	R	39	21	6	6.08	73
Insula	L	-39	15	0	5.29	59
Thalamus	R	6	-24	9	5.53	10
Cingulate	R	9	30	30	6.28	36
**Control_Parametric_ > Pumps_Parametric_**						
Postcentral	L	-66	-18	27	7.30	495
Fusiform	R	39	-6	-33	4.89	15
Middle frontal gyrus	L	-21	18	48	4.45	107
vmPFC	L	-12	33	-15	5.51	58
**Correlation^a^**						
**Negative correlation**						
Insula	R	33	21	0	-4.86	64
Insula	L	-27	21	-3	-5.57	132
Caudate	R	9	6	9	-5.22	65
Caudate	L	-12	6	12	-5.54	63
Anterior cingulate	R	6	39	9	-3.88	45
**Positive correlation**						
Middle temporal gyrus	L	-45	-60	3	7.59	293
Middle temporal gyrus	R	54	0	-24	5.07	64
Medial frontal gyrus	L	-18	39	-12	5.15	111
Culmen	R	15	-36	-24	5.14	58

*R, Right; L, Left; B, Bilateral. ^a^Correlation with mean number of adjusted pumps.*

**Table 3:** Significant clusters of activation in older adults.

Region	L/R/B	*X*	*Y*	*Z*	*t*-value	Cluster size
**Average**						
**Pumps_Average_ > Control_Average_**						
Supplementary motor area, superior frontal gyrus, insula, caudate, putamen	B	-6	0	51	11.20	10387
Lingual gyrus	B	18	-90	-3	11.25	142
Middle temporal gyrus	R	57	-27	-12	3.99	24
Inferior parietal lobule, Precuneus, Middle occipital gyrus	B	48	-45	51	7.42	976
**Control_Average_ > Pumps_Average_**						
Precuneus	B	-3	-54	33	9.22	3330
Middle temporal gyrus	L	-36	24	-27	5.38	409
Middle temporal gyrus	R	60	6	-15	8.32	201
Superior temporal gyrus	R	63	-54	21	5.95	490
Inferior frontal gyrus	L	-45	30	-6	5.27	170
vmPFC	B	-9	57	36	7.89	326
**Parametric**						
**Pumps_Parametric_ > Control_Parametric_**						
Insula	R	33	21	-3	6.51	44
Insula	L	-33	21	-9	7.35	56
Caudate	R	15	6	3	5.08	13
Supplementary motor area, cingulate	B	6	18	51	6.28	30
Lingual gyrus	B	-9	-84	-3	5.09	10
**Control_Parametric_ > Pumps_Parametric_**						
Inferior occipital gyrus	L	-42	-69	-6	6.14	93
Lingual	R	24	-90	-3	5.75	4
Superior parietal lobule	L	-21	-81	45	5.29	25
Precentral gyrus	R	51	-12	54	4.61	7

*R, Right; L, Left; B, Bilateral.*

Age group difference analyses showed that young adults had more activation than older adults in the postcentral gyrus, superior temporal gyrus, middle frontal gyrus, and medial frontal gyrus, whereas no regions were obtained for which older adults had more activation. To further distinguish these age differences, we performed group difference analyses masked separately by positive and negative effect maps. Young adults showed more negative effects (i.e., Control_Average_ > Pumps_Average_) than older adults in the fusiform, bilateral middle occipital lobe, precentral/postcentral gyrus, and a minor positive difference in vmPFC (**Figure 2B**, Blue); no regions were obtained for which older adults had more negative effects than young adults. For positive effects (i.e., Pumps_Average_ > Control_Average_), we found that older adults showed higher activation in the middle frontal gyrus, inferior parietal lobule, middle temporal gyrus, putamen, middle occipital gyrus, and supplementary motor area (SMA) (**Figure 2B**, Red); no regions were observed which evidenced higher activation in young compared with older adults.

#### Neural Activity: Parametric Effects

**FIGURE 3:**
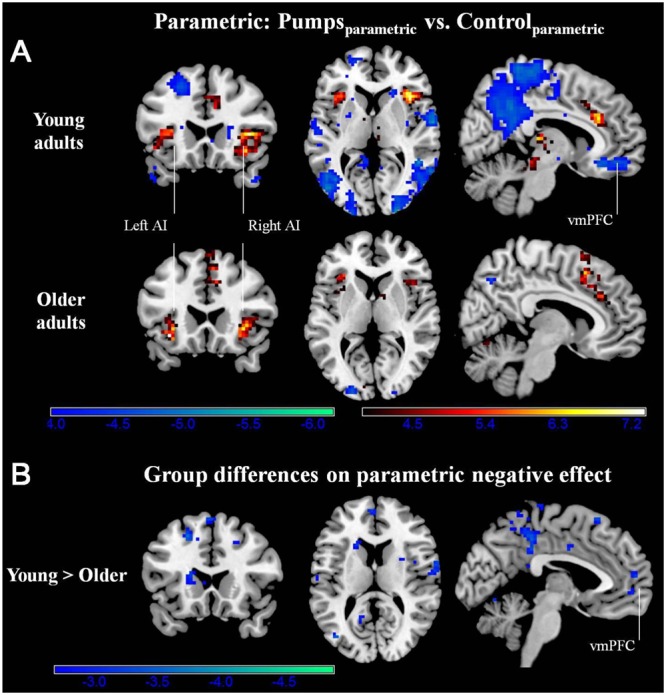
**(A)** Parametric modulation of increasing number of pumps in the young and older age group. The red scale represents Pumps_Parametric_ > Control_Parametric_ and the blue scale represents Control_Parametric_ > Pumps_Parametric_. *p* < 0.05, corrected. **(B)** Age group differences on parametric contrast. The blue scale represents neural regions in which young adults had more negative effect than older adults. *p* < 0.005, uncorrected (scale represents the range of *t*-values). No brain regions were discovered for (1) which older adults had larger negative effects relative to young adults or (2) age group differences on parametric positive effect at this threshold.

We aimed to capture the dynamic nature of risk processing in the BART by estimating the parametric modulation of BOLD responses as a function of the sequentially increasing pumps on reward vs. control balloons (see Schonberg et al., 2012, for a similar analysis). The parametric analysis yielded less widespread neural activity compared to the average pumps contrast described above. Young adults displayed positive effects (i.e., Pumps_Parametric_ > Control_Parametric_) in the bilateral AI, thalamus, and dorsal ACC, and negative effects (i.e., Control_Parametric_ > Pumps_Parametric_) in the fusiform, postcentral gyrus, and vmPFC. Older adults showed positive effects in the bilateral AI, caudate, and SMA, and displayed negative effects in some occipital-parietal regions, but, crucially, no vmPFC areas survived correction (**Figure 3A**; **Tables 2** and **3**, Parametric).

Further, although results from the between-group *t*-tests did not survive whole brain correction, there were voxels in the vmPFC that showed age group differences at *p* < 0.005 uncorrected (**Figure 3B**). The decreasing activity of the vmPFC obtained from the parametric contrast has been suggested to capture value integration in the BART (Schonberg et al., 2012) and the differential pattern of vmPFC parametric activation for young but not older adults suggests that the value integration processes during sequentially increasing pumps is less distinct in older adults compared with young adults. We explored young and older adults’ activation maps at *p* < 0.005 uncorrected to check for differences which may have arisen due to factors such as signal heterogeneity or the small number of subjects in each group. At *p* < 0.005 uncorrected, we observed minor striatal activation in both young and older adults (Supplementary Figure S2), which might be suggestive of some form of reward tracking in the striatum as a function of increasing number of pumps. Interestingly, even at uncorrected level, older adults did not show any vmPFC-related activity, pointing toward genuine age-related differences in vmPFC-related integrative signaling.

#### Regions Correlated with Behavioral Performance

**FIGURE 4:**
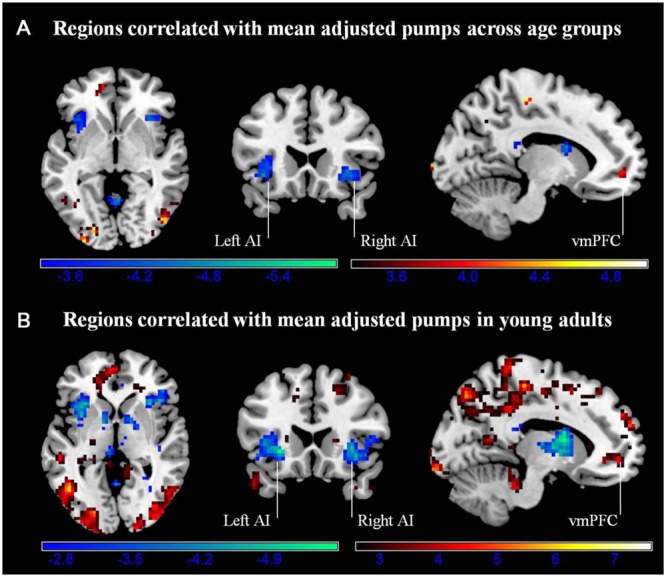
**(A)** Regions correlated with mean adjusted pumps (whole sample). **(B)** Regions correlated with mean adjusted pumps in young adults. Activity obtained from parametric modulation of increasing number of pumps (Pumps_Parametric_ vs. Control_Parametric_) in the bilateral anterior insula (AI) is negatively related to and ventromedial prefrontal cortex (vmPFC) is positively related to participants’ mean adjusted pumps in the young adults. The red scale represents a positive correlation, whereas the blue scale represents a negative correlation, *p* < 0.05, corrected (scale represents the range of *t*-values).

We conducted a whole-brain regression analysis linking a measure of risk taking, mean adjusted pumps in the BART, and neural activity obtained from the “Pumps_Parametric_ vs. Control_Parametric_” contrast. We thus hoped to assess how individual differences in behavioral risk taking were associated with average neural activation patterns. Across age groups, the regression analysis revealed significant negative correlations between participants’ risk taking and activity in the bilateral AI and caudate (**Figure 4A**; **Table 4**). In turn, positive correlations were found between adjusted pumps and activity in the bilateral middle occipital cortex, inferior parietal lobule, and vmPFC. The positive association between behavior and vmPFC activation is reflective of individual differences in the steepness of the predominantly negative slopes observed in the vmPFC: individuals with flatter (i.e., smaller negative effect) slopes on average administered more pumps on cashout balloons compared with individuals with steeper (i.e., greater negative effect) slopes. It is therefore postulated that individuals take more risks on average (i.e., administer more pumps) if the decrease in vmPFC activity is more gradual. Regarding age-related differences, young adults’ regression results were similar to the findings obtained across all individuals, albeit stronger in several regions (**Figure 4B**; **Table 2**, Correlation). However, regression of whole brain activation on mean adjusted pumps for older adults yielded no significant voxels at the correction threshold of *p* < 0.05 and only very sparse association patterns at *p* < 0.005 uncorrected (Supplementary Figure S3).

**Table 4:** Clusters correlated with mean number of adjusted pumps in across age groups.

Region	L/R/B	*X*	*Y*	*Z*	*t*-value	Cluster size
**Negative correlation**			
Insula	L	-36	21	-6	-4.81	83
Insula	R	33	21	-3	-4.52	33
Caudate	B	-12	6	18	-4.62	36
Culmen	B	3	-57	0	-5.89	13
**Positive correlation**			
Inferior temporal gyrus	L	-48	-57	-9	4.58	38
Middle occipital gyrus	R	42	-69	-12	4.75	23
Middle occipital gyrus	L	-21	-84	-3	4.73	62
Inferior parietal lobule	L	-45	-45	45	4.59	36
Medial frontal gyrus	L	-18	45	3	4.19	12
Medial frontal gyrus	L	-12	-27	57	4.60	12

*R, Right; L, Left; B, Bilateral.*

To check that the whole brain regression results were not influenced by outliers and visualize the results with respect to individual differences, additional ROI analyses were conducted on the bilateral AI, bilateral striatum and vmPFC. In particular, mean beta weights were extracted from spheres based on the relevant center coordinates provided by Bartra et al. (2013) to achieve an independent definition of the structures of interest. To note, these analyses are merely for visualizing the relationship between neural activity and performance in the two age groups; the authors acknowledge a degree of circularity when extracting activation from regions identified by whole brain analyses as being associated with performance. However, given that no significant association was obtained from the whole brain analyses for older adults, we were interested in visualizing the distribution of performance against activity in both age groups.

**FIGURE 5:**
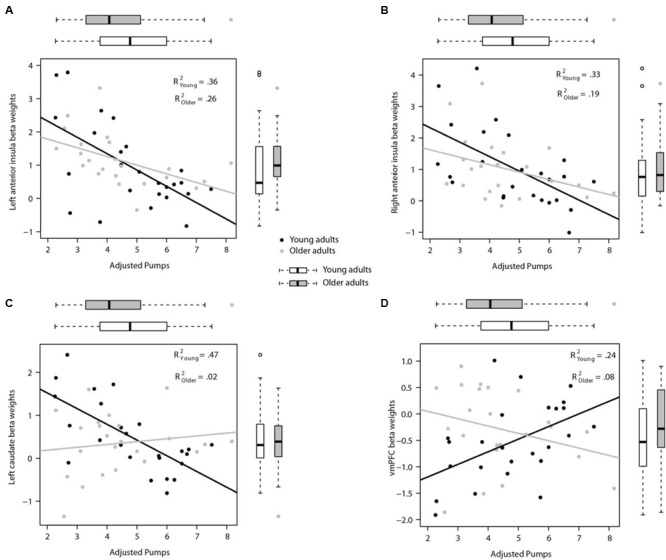
**Region of interest (ROI) analyses for links between individual neural and behavioral differences. (A)** Participants’ mean adjusted pumps negatively correlated with their BOLD signals in the left AI and **(B)** the right AI for both young and older adults. **(C)** Activity in the left striatum was significantly correlated with mean adjusted pumps in young adults, but not in older adults. **(D)** Activity in the vmPFC was positively correlated with mean adjusted pumps in young adults, but not in older adults. The boxplots on top of the plot show the distribution of mean adjusted pumps in the young and older age group, respectively, whereas boxplots to the left of the plot show the distribution of the signal changes in the left/right AI, vmPFC and left striatum, respectively. ROIs were created as 4 mm radius spherical regions covering bilateral anterior insula and left striatum, and an 8 mm radius spherical region in the vmPFC (center coordinates based on meta-analysis by Bartra et al., 2013).

Activity in the seed region of the left AI during Pumps_Parametric_ vs. Control_Parametric_ was negatively correlated with adjusted pumps in young (*r* = -0.60, *p* < 0.01), and older adults (*r* = -0.51, *p* < 0.05; *Z* = 0.42, *p* > 0.05; **Figure 5A**). A comparable pattern was found in the right AI, with older adults showing a correlation between adjusted pumps and brain activity that was similar to that found for young adults (*r* = -0.44, *p* < 0.05, *r* = -0.58, *p* < 0.01, respectively; *Z* = 0.62, *p* > 0.05; **Figure 5B**). These findings merely visualize the whole brain regression results, suggestive of comparable insular tracking of potential loss (uncertainty) in older and young adults. In addition, extracted beta weights from the left (but not right) striatum correlated negatively with mean number of adjusted pumps in young (*r* = -0.68, *p* < 0.001) but not older adults (*r* = 0.15, *p* = 0.50; **Figure 5C**); the difference between these two correlations was significant (*Z* = 3.17, *p* < 0.01). As expected from the whole brain analyses, activation in the vmPFC was positively correlated with adjusted pumps in young adults (*r* = 0.48, *p* < 0.05), but not in older adults (*r* = -0.22, *p* = 0.31; **Figure 5D**); the difference between these two correlations was significant (*Z* = 2.42, *p* < 0.05). We obtained comparable results when using masks derived from peak contrast coordinates and varying radii. To note, although occupying a similar range, the distribution of mean beta weights extracted from the parametric modulation of vmPFC activity in older adults appears positively skewed compared with a relatively more normal distribution for young adults (**Figure 5D**). In contrast, the distribution of extracted mean activation slopes for the insula and striatum is relatively more similar in older and young adults.

Taken together, these results suggest that although neural representations of reward and risk as well as the tracking thereof remain relatively stable across age groups, their predictive validity for behavior may be different for young and older adults. Moreover, older adults’ tracking of value in the vmPFC was different from that of young adults, also manifested by the differential vmPFC activation profiles and predictive validity of vmPFC activation for mean pumping (i.e., risk taking) behavior in the BART.

## Discussion

The present study investigated adult age differences in behavior and neural activations associated with the BART, a widely used naturalistic risk-taking task (Lejuez et al., 2002). Specifically, we asked young and older adults to undergo fMRI while completing a version of the BART consisting of different types of balloons, which either did (experimental) or did not (control) involve monetary risks and rewards. The different balloon types were leveraged to build contrasts that captured the neural signatures associated with young and older adults’ risky decision-making processes (Rao et al., 2008; Schonberg et al., 2012; Helfinstein et al., 2014).

Our results indicate considerable similarity between young and older adults in the behavioral outcomes of the BART, including similar average number of pumps per balloon for the two age groups. Older adults were, however, more likely to cashout their temporary wins relative to young adults, potentially indicating higher levels of risk-aversion with increased age (Mata et al., 2011; Best and Charness, 2015). Overall, these behavioral outcomes contribute to the heterogeneity of findings concerning age differences in the BART (Henninger et al., 2010; Cavanagh et al., 2012; Rolison et al., 2012).

Concerning our neuroimaging results, we replicated past findings with young adult samples suggesting a link between neural activation and the processing of risk and reward. Specifically, using contrasts between neural activation while pumping in experimental relative to control balloons in the BART, we found significant neural activations in the caudate, bilateral insula, and parietal regions, as well as in the vmPFC, which are comparable with previous findings (Rao et al., 2008, 2014; Schonberg et al., 2012). Also consistent with a previous study that analyzed parametric neural activation as a function of increased exposure to risk and rewards, we found that vmPFC activity decreased whereas bilateral AI activity increased as participants pumped up each balloon (Schonberg et al., 2012). Concerning age differences, group average comparisons identified similar patterns of activations in the striatum and AI as well as deactivation in the vmPFC in both age groups. Our findings are in line with previous studies showing intact representation of reward (Samanez-Larkin et al., 2007, 2014) and loss anticipation (Samanez-Larkin et al., 2008; but see Samanez-Larkin et al. (2007) for altered insular sensitivity during loss anticipation). The lack of differences between young and older adults in ventral striatal activation during gain anticipation may imply that the ventral striatal regions may not be as compromised by age as are the neural substrates recruited in reward reversal learning tasks, such as the PFC regions (Marschner et al., 2005; Samanez-Larkin et al., 2007). Some differences between young and older individuals were observed for the average contrasts: the comparatively lower deactivation/higher activation for risky vs. control balloons in older adults may suggest systematic differences in the neural representation of value-related processes, for instance slightly higher sensitivity to gains (higher striatal activation) or weaker integration (less vmPFC deactivation). It is noteworthy that some of the regions for which age differences were observed in the average contrast analysis overlap with regions engaged in the default mode network (Raichle et al., 2001) and brain networks identified for working memory tasks (Tomasi et al., 2006). Consequently, it is also possible that the few differences observed for average contrasts stem from older adults dealing differently with the process of being engaged in and completing a task with some memory demands.

In contrast, parametric analyses at group level found that young and older adults evidenced similar tracking of pumps in the AI, but only young adults showed parametrically decreasing activity in the vmPFC. Interestingly, strong striatal activation might be expected as a function of parametric pumps, given that the striatal coding of gains (cf. Tom et al., 2007) ought to be reflected in the parametric tracking of pumps, the latter being a potential proxy for increasing gain on a given trial in the BART. The absence of a strong striatal signal in this study as well as in the study by Schonberg et al. (2012) is likely to be reflective of increasing pumps being processed not as increasing gain, but as increased risk of loss. Against a backdrop of work that has assigned the processing of risk to the insula (Volz et al., 2003; Kuhnen and Knutson, 2005; Preuschoff et al., 2008; Samanez-Larkin et al., 2008), our parametric results further support findings from the average contrasts, speaking to unaltered insula-based tracking of increasing risk in old age.

The combination of relatively preserved insula signaling and age-related differences in vmPFC signaling in response to increasing risk observed from the parametric analyses support the notion of the vmPFC as a platform for integration and convergence of information (Schonberg et al., 2012; Bartra et al., 2013; Clithero and Rangel, 2014; Halfmann et al., 2014, 2016). Specifically, we propose that with age, individuals may attach different weights to different aspects of a decision context, or alternatively, are less consistent across time in the weights attached to particular options. Put differently, although older and young individuals in the current study responded with comparable risk and reward signals, the two groups differed with respect to the integration of risk and reward into a subjective value signal. In support of this line of argument, past theoretical and empirical work converges on the idea that value representations are affected by age-related anatomical and/or functional differences. Anatomically, there is a global declining of gray matter volume in the prefrontal cortex (PFC) with age (Raz et al., 1997), a thinner cortical thickness of left vmPFC (Cassidy and Gutchess, 2012), and a decreasing white matter integrity in thalamocorticostriatal paths, which run from the thalamus to the medial PFC and from the medial PFC to the ventral striatum (Samanez-Larkin et al., 2012). Functionally, impaired integration processes from the vmPFC may arise from less effective coding by single systems or degrading glutamatergic projections from the medial PFC to the striatum (Samanez-Larkin and Knutson, 2015). Recent work by Halfmann et al. (2014, 2016) linked reduced vmPFC signaling to disadvantageous decision patterns in the Iowa Gambling Task, which the authors interpreted as support for the notion of noisier value representation in older adults (Li et al., 2007; Samanez-Larkin et al., 2010). This view is also consistent with previous studies showing age-related reductions in activity during learning from rewards in the vmPFC but not during learning from monetary losses in the insula and striatum (Eppinger et al., 2013). Bridging the gap between the neural and the behavioral level, it is conceivable that a decreased signal-to-noise ratio in older adults may in part be underlying the mixed behavioral patterns obtained in past work using the BART (Henninger et al., 2010; Cavanagh et al., 2012; Rolison et al., 2012): different experimental implementations of the BART may rely on more (or less) efficient integration of information, hence decision outcomes are perhaps affected differently by an integration process that is subject to age-related changes. Although the current study cannot offer direct evidence supporting this suggestion, the notion of heterogeneity in study results being linked to brain signal heterogeneity offers a potential avenue for research aimed at connecting age-related neural and behavioral differences in decision-making tasks.

Current theories emphasize the contribution of both cognitive and affective processes to age differences in decision making (Samanez-Larkin and Knutson, 2015; Schiebener and Brand, 2015). Our results, however, indicate that what could be potentially considered affective components, such as neural coding of risk in the AI and reward in the striatum, are relatively preserved with aging. In turn, value coding and integration in the vmPFC seems less robust. Whether such changes can be deemed the result of cognitive or affective components is unclear. The absence of both a consistent group level value signal and a correlation with behavior in the vmPFC in our sample of older adults for instance may result from older adults exhibiting potentially noisier intra-individual (e.g., Samanez-Larkin et al., 2010) or more heterogeneous inter-individual coding of value in this region, suggesting a more cognitive explanation. Alternatively, given that we find older adults’ risk-taking behavior to be linked with insula more so than with vmPFC signaling in response to increasing risk, there may also be motivational components associated with the relative importance or attention devoted to gains and losses (Mata and Hertwig, 2011).

The exact mechanisms underlying age differences in value coding and integration in the vmPFC are still to be identified. Future work using the BART could contribute to clarifying these issues by manipulating task characteristics, such as reward structure and loss probability, to better tease apart the contribution of neural risk and reward signals in young and older adults to an overall utility signal coded in the vmPFC. Future work may also want to directly test the role of anatomical and functional deficits in and between medial prefrontal and other brain regions by using behavioral performance indices in voxel-based morphometry (e.g., Strenziok et al., 2011; Peper et al., 2013; Gilaie-Dotan et al., 2014), diffusion tensor imaging (e.g., Kwon et al., 2014; Van den Bos et al., 2014; Leong et al., 2016), or effective connectivity analysis (e.g., Hare et al., 2014).

With respect to limitations, risk and reward were directly correlated in the current BART version hence it was not possible to dissociate risk from reward through parametrically altering each decision component. However, given the comparatively rich pool of studies that have investigated risky decision making as well as the impact of aging thereon, the advantage of using a task that offers external validity outweighs many of its shortcomings. As alluded to above, future work is required which tries to dissociate reward from risk as well as reward and risk from subjective value. We are currently in the process of answering this call to uncover differential sensitivity to risk or rewards as a function of age. Further, future studies should strive to collect data from lifespan samples to account for intra- as well as inter-individual change to derive neural and behavioral trajectories of risk taking across the full range of the adult lifespan.

## Conclusion

To conclude, our comparison of young and older adults’ neural activation during decision making in the BART suggests that the two age groups show similar patterns of activation in the AI, possibly coding for the probability of loss, yet differ in the recruitment of the vmPFC, which is thought to subserve value integration and representation. Our results suggest that the integration of risk and reward resulting in overall utility representations may be affected by aging. Our results show the need for distinguishing different neural components underlying risk taking, including the processing of risk, rewards, and the integration of the two, to uncover possible differences in risk taking across the lifespan.

## Author Contributions

JY conceived the idea, designed the study, analyzed and interpreted data, drafted the manuscript. RM and LM interpreted data, and participated in writing up and revising the manuscript. XL and LF assisted to analyze and interpret data.

## Conflict of Interest Statement

The authors declare that the research was conducted in the absence of any commercial or financial relationships that could be construed as a potential conflict of interest.

## Footnotes

1 http://www.fil.ion.ucl.ac.uk/spm

2 http://www.alivelearn.net/xjview8
