# Supplementary material for: Altered Value Coding in the Ventromedial Prefrontal Cortex in Healthy Older Adults
Source: Front Aging Neurosci. 2016 Aug 31;8:210. doi: 10.3389/fnagi.2016.00210 (PMC5005953; doi:10.3389/fnagi.2016.00210)
Supplement: Supplementary file 1 [file Image_1.PDF]

Supplementary Figures

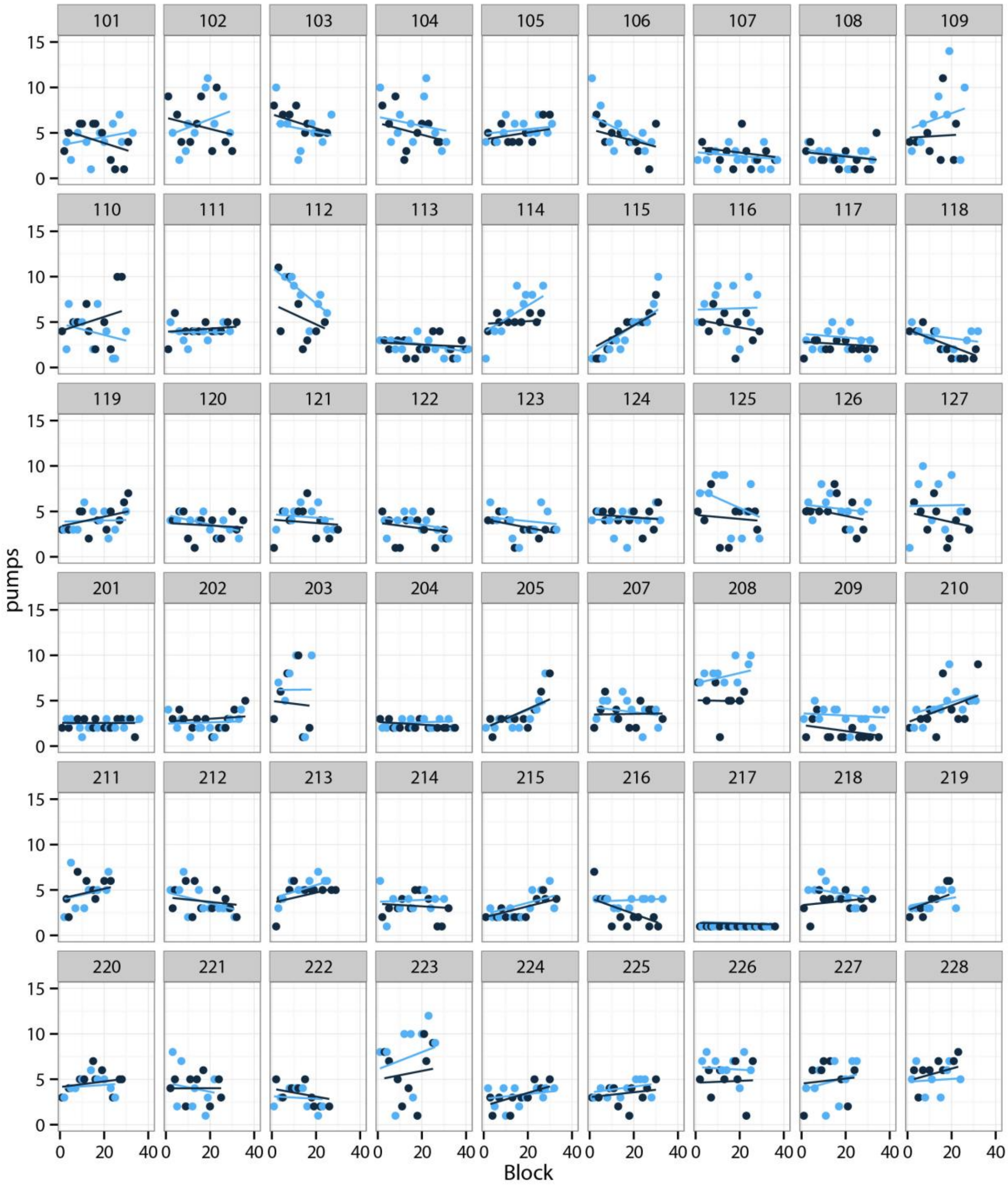

Figure S1. Number of pumps per balloon for each participant as a function of trial number. Participant numbers 101~127 represent older adults, 201~228 represent young adults. Light blue dots represent the number of pumps for high-capacity balloons, and dark blue dots represent the number of pumps for low-capacity balloons. Lines represent fits of mixed-effect modeling on pumps in which effects of balloon type and trial are allowed to vary by participant.

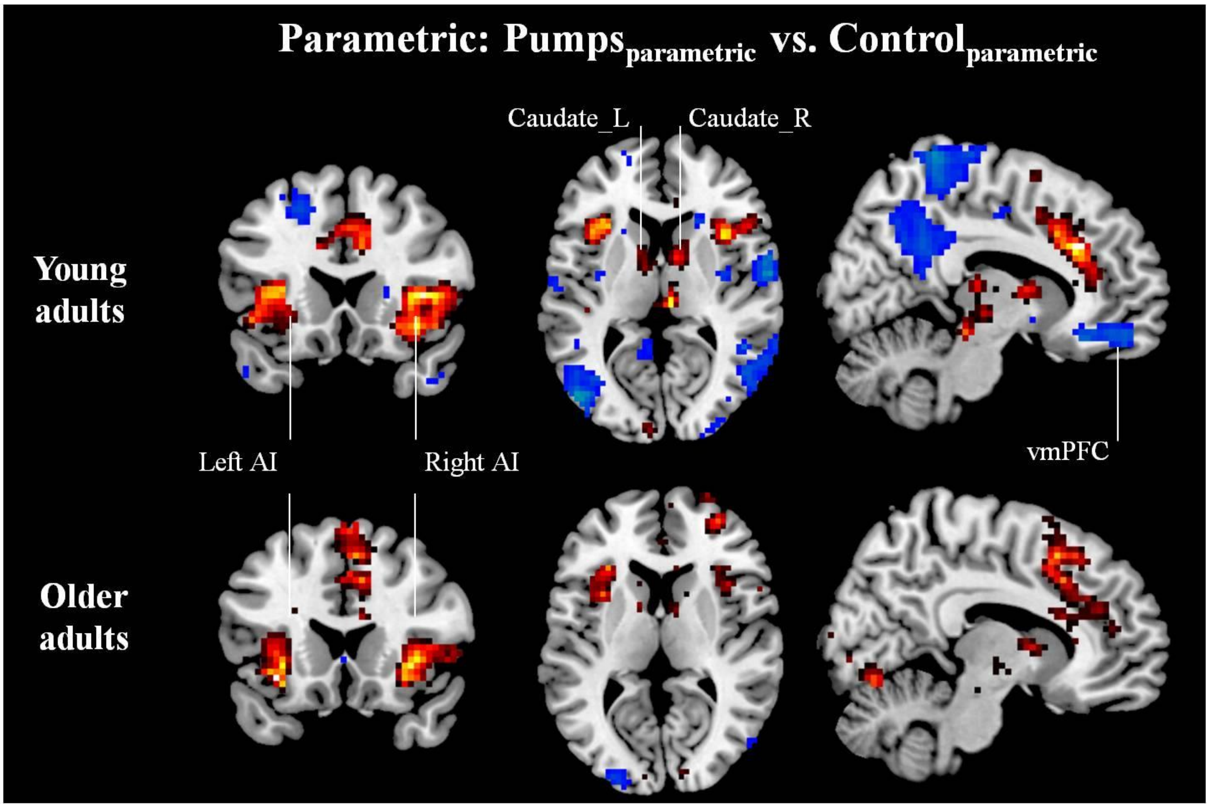

Figure S2. Replication of Figure 3 (Panel A), at p < .005 uncorrected. A) Parametric modulation of increasing number of pumps in the young and older age group. The red scale represents $\text{Pumps}_{\text{Parametric}}$ > $\text{Control}_{\text{Parametric}}$ and the blue scale represents $\text{Control}_{\text{Parametric}}$ > $\text{Pumps}_{\text{Parametric}}$.

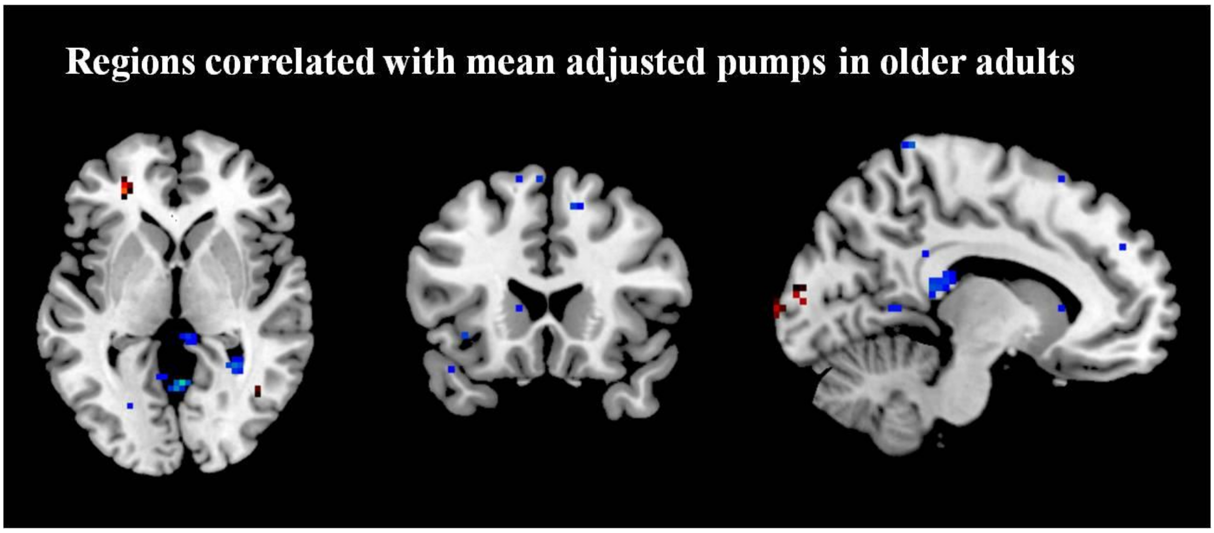

Figure S3. Replication of Figure 4 for older adults, at $p < .005$ uncorrected. Regions correlated with mean adjusted pumps in older adults.
